# Cultural adaptation of a self-help app for grieving Syrian refugees in Switzerland. A feasibility and acceptability pilot-RCT

**DOI:** 10.1016/j.invent.2025.100800

**Published:** 2025-01-18

**Authors:** Anaïs Aeschlimann, Eva Heim, Clare Killikelly, Nesrin Mahmoud, Farhad Haji, Rilana Tanja Stoeckli, Monia Aebersold, Myriam Thoma, Andreas Maercker

**Affiliations:** a Department of Psychology, University of Zurich, Zurich, Switzerland; b Institute of Psychology, University of Lausanne, Lausanne, Switzerland; c Swiss Red Cross Bern, Bern, Switzerland; d Institute of Psychology, University of Bern, Bern, Switzerland

**Keywords:** Cultural adaptation, Refugees, Bereavement, Mobile mental health, Syrian, Self-help

## Abstract

**Background:**

The global refugee population has significantly increased, with Syrian refugees being one of the largest displaced groups. Bereavement represents a major challenge. However, access to mental health care is limited by structural and cultural barriers. Internet-based interventions (IBIs) offer a promising solution, but most are developed in Western contexts, limiting their cultural relevance. This study aimed to evaluate the acceptability, feasibility, and preliminary efficacy of a culturally adapted self-help IBI for bereaved Syrian refugees in Switzerland.

**Methods:**

In a mixed-methods pilot randomized controlled trial (RCT), 30 bereaved Syrian refugees were randomly assigned to a 5-week app-based intervention or a waitlist control group. Semi-structured interviews with the intervention group provided qualitative insights on feasibility and acceptability and were analyzed with framework analysis. Quantitative data assessed treatment satisfaction, adherence, and preliminary efficacy on grief, depression, posttraumatic stress disorder (PTSD), anxiety, well-being, disability, post-migration difficulties, and social support. Descriptive statistics were used for feasibility and acceptability, while linear mixed-effects models assessed efficacy.

**Results:**

High treatment satisfaction, a low dropout rate and adherence of 40 % were found. Qualitative interviews indicated the intervention was relevant and beneficial, further adaptations were suggested. No significant group differences were found on bereavement or secondary outcomes. However, trends indicated reduced grief, anxiety, PTSD, and depression, with improved well-being and social support in the intervention group.

**Conclusions:**

The results suggest that this culturally adapted IBI is both feasible and acceptable for Syrian refugees. While trends are promising, a larger RCT is needed to investigate efficacy. This intervention shows potential as meaningful support for bereaved Syrian refugees.

## Highlights

• A culturally adapted internet-based self-help intervention for bereaved Syrian refugees was evaluated.
• A mixed-methods pilot-RCT was conducted with an intervention group and a wait-list control group.
• Results indicated that the intervention was found to be highly feasible and acceptable.
• Findings on preliminary efficacy are promising but non-significant and need to be confirmed in a fully powered-RCT.

## 1 Introduction

Over the past decade, the global refugee population has soared to 43.4 million, a threefold increase driven by escalating conflicts, persecution, and humanitarian crises worldwide (UNHCR, 2024). Among the largest displaced groups are Syrian refugees, with 13.8 million people forcibly displaced — representing the highest proportion of displaced individuals relative to any national population globally (UNHCR, 2024). After more than thirteen years of civil war, Syrians remain in urgent need of support, with 16.7 million requiring humanitarian assistance as of 2024, the highest level since the conflict began (United Nations, 2024). The war has claimed 306,887 civilian lives between 2011 and 2021, though the true toll is likely higher due to indirect casualties and unreported cases (Office of the United Nations High Commissioner for Human Rights, 2022). In Switzerland, Syrian refugees represent the second-largest refugee population, with over 14,000 granted refugee status by mid-2024, and an additional 6635 awaiting asylum decisions (Staatssekretariat für Migration, 2024a, Staatssekretariat für Migration, 2024b).

Refugees experience high rates of mental health issues such as anxiety, depression, and posttraumatic stress disorder (PTSD), with studies reporting prevalence rates of 33 % among Syrian refugees in high-income Western countries (Nguyen et al., 2022) and 47 % in Switzerland specifically (Kiselev et al., 2020b). A central mental health issue for victims of armed conflicts, such as Syrian refugees, is the grief resulting from the death of loved ones (Hassan et al., 2016). In a study with refugees in Germany, Comtesse and Rosner (2019) found that 92 % had lost a loved one. This is reflected in significantly higher rates of Prolonged Grief Disorder (PGD) among refugees, with a pooled prevalence of 33.2 %, compared to 3.3 % to 4.2 % in the general population (Kokou-Kpolou et al., 2020; Lechner-Meichsner et al., 2024; Rosner et al., 2021).

Refugees face several factors that may exacerbate their grief, making them a particularly vulnerable group. These factors include the often-traumatic circumstances surrounding their losses and the multiple challenges associated with post-migration (e.g., legal status; Djelantik et al., 2020a, Djelantik et al., 2020b; Kokou-Kpolou et al., 2020; Lechner-Meichsner et al., 2024). Post-migration stressors, such as legal challenges, language barriers, and social isolation, have also been linked to adverse mental health outcomes, including depression, anxiety, and PTSD, in refugee populations (Kiselev et al., 2020a; Schick et al., 2016). Additionally, the inability to partake in culturally significant mourning rituals may complicate the grieving process, while the disruption of their social networks may lead to diminished social support, potentially further compounding their grief (Lechner-Meichsner et al., 2024; Wojtkowiak et al., 2021).

Individuals experiencing clinically significant grief who do not receive appropriate support may face not only the adverse psychosocial effects associated with their grief symptoms but also a range of additional serious health and psychosocial complications. These challenges include heightened risks of cardiovascular problems, elevated blood pressure, substance misuse, harmful health behaviors, and suicidal tendencies (Fujisawa et al., 2010; Kersting et al., 2011; Maercker et al., 2008; Prigerson et al., 2009). Despite the pressing need for bereavement support, a large mental health treatement and support gap exists among refugees in host countries (Dumke et al., 2024; Satinsky et al., 2019). This gap is largely attributable to various structural and socio-cultural barriers, including language barriers, limited resources, mistrust towards healthcare providers, and concerns about stigma (Kiselev et al., 2020a).

Therefore, it is critical to develop accessible interventions that can bridge this gap in mental health care for refugees. One promising approach is the use of internet-based interventions (IBIs), such as self-help apps, which offer low-threshold, easily accessible psychological support, potentially reduce stigma, and show promise for cost-effectiveness (Schröder et al., 2016). Supporting this approach, the American Psychological Association advocates for scaling individual interventions through innovative platforms (Dodge et al., 2024), and the World Health Organization (WHO, 2018) highlights IBIs' ability to reach underserved groups, including refugees. Syrian refugees, in particular, are well-suited for IBIs due to their widespread use of smartphones and internet access (Maitland and Xu, 2015; Rutkin, 2016). Previous implementations, such as the Step-by-Step app for depression, have shown these tools to be effective, feasible, and acceptable within this population (Cuijpers et al., 2022; Heim et al., 2021b).

Systematic reviews and meta-analyses have demonstrated the effectiveness of IBIs in helping individuals cope with bereavement (Wagner et al., 2020; Zuelke et al., 2021). However, these interventions, much like bereavement interventions in general, have predominantly been developed and implemented in Western settings, which limits their relevance for non-Western populations, such as Syrian refugees (Aeschlimann et al., 2024b). Furthermore, this recent review (Aeschlimann et al., 2024b) identified only two culturally adapted face-to-face bereavement interventions specifically designed for refugee populations, highlighting a significant gap in the field. Grief and the way it is experienced and expressed, as well as mourning rituals and practices, are deeply influenced by culture (Rosenblatt, 2008). To ensure that bereavement support is meaningful and effective for these individuals, it is essential that IBIs be culturally adapted to align with their experiences of grief and loss. Cultural adaptation has been shown to significantly improve the effectiveness of mental health interventions for non-Western populations, with numerous reviews indicating that adapted approaches are generally more effective, acceptable, and may increase adherence compared to non-adapted ones (Arundell et al., 2021; Benish et al., 2011; Chowdhary et al., 2014; Griner and Smith, 2006; Hall et al., 2016; Harper Shehadeh et al., 2016; Naeem et al., 2019).

To the best of our knowledge, no culturally adapted IBI specifically targeting bereavement among Syrian refugees has been developed, highlighting a significant gap in both the literature and mental health care provision. In response, a culturally adapted self-help app offering bereavement support for Syrian refugees was created in a collaboration between the University of Zurich (UZH) and the Swiss Red Cross (SRC). This intervention was rigorously adapted using a bottom-up approach, following the Reporting Cultural Adaptation in Psychological Trials (RECAPT) guidelines (Heim et al., 2021a). The complete documentation of this adaptation process is provided in a separate publication (Aeschlimann et al., 2024a).

Conducting a pilot study with a small sample size is an essential step in the development and evaluation of psychological interventions (Onken et al., 2014; Thabane et al., 2010). In this mixed-methods pilot RCT, the primary objective was to assess the acceptability and feasibility of a culturally adapted, 5-week, unguided self-help app designed for grieving adult Arabic-speaking Syrian refugees (see description below). Additionally, we aimed to investigate preliminary treatment effects in an exploratory manner as a secondary objective.

## 2 Methods

The description of the study adheres to the reporting format outlined in the Consolidated Standards of Reporting Trials (CONSORT) 2010 guidelines (Schulz et al., 2010)), including the extension for pilot and feasibility studies (Eldridge et al., 2016). See Appendix A for the completed checklists. This pilot-RCT was registered in ClinicalTrials.gov (NCT06246708) and the Swiss National Clinical Trials Portal (SNCTP000005760) in January 2024. It adhered to the ethical principles of the World Medical Association Declaration of Helsinki (2013), the ICH-GCP guidelines (International Conference on Harmonization, 2016), and the applicable local legal requirements. The procedures were approved by the Cantonal Ethics Committee Zurich (ID: BASEC-Nr. 2023–01959).

### 2.1 Design

The present study was a two-arm mixed-methods pilot-RCT with an active intervention group (self-help app) and a waitlist control group, with participants randomly allocated in a 1:1 ratio. The waitlist group gained access to the self-help app after post-measurement (T1). The study included baseline assessment (T0) and post-measurement including a qualitative evaluation (T1, five weeks post-randomization).

### 2.2 Sample and recruitment

Participants were recruited by the study team at the UZH and the SRC. Recruitment occurred in person at asylum centers, through previous study participants (Aeschlimann et al., 2024a), and snowball sampling, from January to June 2024 across Switzerland. Advertisements were posted on websites, social media, and forums. Additionally, SRC specialists and cantonal asylum centers promoted recruitment, with many participants recruited by Syrian-origin study translators. Inclusion criteria were: (1) Syrian refugees in Switzerland, (2) Bereavement experience at least three months prior, (3) A minimum score of 3 on at least one item of the International ICD-11 Prolonged Grief Disorder Scale (IPGDS; Killikelly and Maercker, 2018), (4) Smartphone ownership with internet access, (5) Proficiency in Arabic (reading, writing and understanding), and (6) Ability to provide informed consent. A low cut-off score for grief severity and time since death was applied to reflect the implementation and accessibility strategy of the SRC, ensuring the intervention is available to a broad range of individuals seeking grief support, irrespective of formal diagnostic criteria. Exclusion criterion was acute suicidality. Participants received a 50-CHF supermarket coupon for completing each of the T0 and T1 assessments. Public transport users were reimbursed with supermarket coupons equivalent to their ticket value (up to 40 CHF for both appointments).

### 2.3 Procedure

Interested individuals received detailed study information, and screening for inclusion criteria was conducted (informed consent was required for the IPGDS and completed at T0). Screening was conducted either in person or via phone, based on participants' preferences. Those meeting the criteria were invited for the baseline assessment (T0), while those ineligible received mental health support contacts. All assessments occurred in person at UZH, SRC in Bern, or another suitable location chosen by the participant. An Arabic-speaking team member or a German-speaking researcher with a Syrian interpreter conducted appointments, lasting 1–2 h depending on translation needs. The interviewers were unacquainted with the participants, but the interpreters knew several participants.

At T0, participants provided written informed consent before completing the baseline questionnaire. After written informed consent was obtained, the first part of the baseline questionnaire up until the IPGDS was administered. If they met the final inclusion criterion, they proceeded with the full questionnaire. Participants were then randomly allocated to conditions in a 1:1 ratio using a sequence generated by an independent statistician and concealed from the study team, implemented via the REDCap® randomization module (Harris et al., 2009). Blinding of participants and research team members was not possible due to the waitlist control design. After informing them of their allocated group and the next steps, a second appointment for T1 (five weeks) later was arranged. Participants also received mental health support contacts in case of a crisis. For participants in the waitlist control group T0 was completed at this point. Participants in the intervention group were assisted in downloading the self-help app and creating a user account, given a short instruction and were encouraged to complete one chapter of the app per week.

At T1 all participants completed the same questionnaire battery as for T0. The intervention group completed an additional questionnaire on intervention satisfaction and a brief semi-structured interview regarding app feasibility and acceptability. Interviews were audio-recorded and transcribed in German, capturing both the interviewer's direct input and the interpreter's real-time translations, using MAXQDA 2024 (VERBI Software, 2024). Each interview lasted 13 to 58 min. After T1, the control group received app access with an introduction.

A study phone was operated by an Arabic-speaking team member to provide information, reschedule appointments, and assist with technical issues.

### 2.4 Conditions

#### 2.4.1 Treatment group

Participants in the treatment group received access to an unguided self-help app, aimed at providing culturally sensitive support to grieving Syrian refugees in Switzerland (Aeschlimann et al., 2024a). Developed in collaboration with the SRC, the app is intended to serve as a supplementary psychological module for the Sui app (“Selbsthilfe - Unterstützung - Information” [Self-help - Support - Information]), an IBI for psychosocial support for refugees (Stoeckli et al., 2024).

The app is available in Arabic and German on iOS, Android, or via a web browser. It includes an introduction and five main sessions addressing different topics (e.g., psychoeducation about grief reactions, social network, rituals, etc.) drawing on approaches including mindfulness and body-related techniques, cognitive restructuring and self-compassion, among others (for more details see Aeschlimann et al., 2024a). Two additional sessions provide technical help and mental health resources. New sessions were unlocked every 4 days, with participants encouraged to complete one session per week.

The app includes psychoeducational texts, illustrations, interactive exercises, audio-exercises and video-testimonials. A fictional bird character, “Sui”, guides users through the app, offering, explanations, encouragement and reminders for breaks. Vignette stories featuring fictional Syrian refugees, Yasmin and Amir, illustrate how to use the app and engage in its exercises, enhancing user motivation and relatability. Users can personalize the app by saving favorites, setting reminders, and using a diary feature to track their thoughts and emotions.

#### 2.4.2 Waitlist control group

Participants in the waitlist control group received access at T1, five weeks after baseline assessment.

### 2.5 Measures

All measures used in this study were validated Arabic translations previously applied in research involving Syrian refugees (e.g., Harper Shehadeh et al., 2020; Heim et al., 2021a, Heim et al., 2021b). Full descriptions of the measures are available in Appendix B. Socio-demographic data were collected only at T0, and variables related to feasibility and acceptability were collected only in the intervention group at T1. All other measures were collected across both conditions at T0 and T1.

#### 2.5.1 Feasibility and acceptability

Treatment satisfaction was assessed using the Client Satisfaction Questionnaire for Internet Interventions (CSQ-I; Boß et al., 2016). Qualitative feedback on feasibility and acceptability was gathered through a semi-structured interview (see Appendix C) based on Kallio et al. (2016), the RECAPT criteria and previous project phases (Aeschlimann et al., 2024a). Adherence was measured via app usage completion percentages.

#### 2.5.2 Preliminary efficacy

Grief severity was measured using the International ICD-11 Prolonged Grief Disorder Scale (IPGDS; Killikelly and Maercker, 2018). Secondary outcomes included depression symptoms Item (PHQ-9; Kroenke and Spitzer, 2002), PTSD symptoms (PCL-5; Price et al., 2016), anxiety symptoms (GAD-7; Spitzer et al., 2006), health and disability levels (WHODAS 2.0; Üstün et al., 2010), and psychological well-being (WHO-5; Bech et al., 2003).

#### 2.5.3 Further outcomes

Post-Migration Living Difficulties (PMLD; Schick et al., 2016) and perceived social support (MSPSS; Zimet et al., 1988) were also measured.

### 2.6 Sample size

Our primary aim was to investigate feasibility and acceptability of the intervention. As a pilot study with a small sample size, the design was exploratory and lacked the statistical power to detect significant differences. No formal sample size calculation was conducted due to the study's pilot nature. However, we estimated the sample size to be 20–25 % of what would be required for a fully powered RCT. To detect a moderate effect size (*d* = 0.50) with 80 % power at a two-sided alpha of 0.05, a future RCT would need 128 participants. We therefore aimed to recruit 30 participants for this pilot RCT, a commonly recommended sample size for initial feasibility assessments and preliminary insights into treatment effects (Lancaster et al., 2004).

### 2.7 Data analysis

#### 2.7.1 Qualitative analysis

All qualitative data were analyzed using framework analysis, a structured and flexible approach for managing and examining large qualitative data sets, particularly for analyzing data on similar topics (Gale et al., 2013). This method produces organized outputs of summarized data (framework matrix), with themes emerging through comparisons within and between interviews. Both inductive and deductive coding were employed, with deductive codes developed in MAXQDA 2024 (VERBI Software, 2024) based on the RECAPT criteria and the framework applied in our previous study (Aeschlimann et al., 2024a). Each transcript was independently coded in parallel by two coders to ensure consistency and enhance reliability (MacPhail et al., 2016). Coders engaged in iterative discussions to refine the coding framework and resolve discrepancies, ensuring consensus and methodological rigor. Data saturation was not formally calculated but was informed by prior research, which suggests that 9–17 interviews are typically sufficient to achieve saturation in qualitative studies (Hennink and Kaiser, 2022). Based on this, we adopted a pragmatic approach and included all 15 participants in the treatment condition in our qualitative analysis. The research team brought diverse professional and cultural perspectives to the qualitative analysis. The two coders included a Swiss PhD candidate with prior experience analyzing qualitative data on grief among Syrian refugees, expertise in cultural adaptation, and a strong background in qualitative methodologies, and an Iranian-Swiss master's student in psychology with experience providing mental health support to refugee populations in Switzerland. To address potential influences of positionality, the coding framework was refined through iterative discussions and collaboration, ensuring methodological rigor. Additionally, Syrian-origin interpreters were consulted to provide cultural clarification and enhance the relevance of the analysis.

#### 2.7.2 Quantitative analysis

Statistical analyses were performed using R version 4.4.1 (R Core Team, 2024) with a significance threshold of *p* < 0.05. Effect sizes (Cohen's *d*) with 95 % confidence intervals were reported for all outcomes. Normality and homogeneity of variance were confirmed using the Shapiro-Wilk and Levene's tests, respectively. Baseline comparisons between treatment groups employed *t*-tests for continuous variables and Chi-squared tests for categorical variables.

To evaluate feasibility and acceptability, descriptive statistics assessed dropout rates, adherence, and treatment satisfaction. Given the exploratory design and limited statistical power, the analyses focused on efficacy were not aimed at detecting significant differences but were instead considered exploratory. An Intention-to-Treat (ITT) analysis was conducted. Due to missing data, linear mixed-effects models (LMM) replaced the originally planned mixed-effects ANOVA, offering greater robustness. LMMs evaluated the fixed effects of time (baseline vs. post-assessment), group (intervention vs. waitlist control), and their interaction (time × group) on primary and secondary outcomes, with random intercepts for participants. Dummy coding was applied, with the waitlist group as the reference.

Control variables (age, sex, CSQI, adherence, PMLD at T0, loss type) were included in follow-up models to assess moderating effects. Only significant changes or three-way interactions involving control variables are reported. Models were fitted using the nlme R package (Pinheiro et al., 2024), and missing data were handled through maximum likelihood estimation (Graham, 2009). Corrections for multiple testing were avoided due to the small sample size (Nakagawa, 2004), but effect sizes and confidence intervals were provided for context.

## 3 Results

### 3.1 Participant flow, baseline characteristics and participant contacts

**Fig. 1:**
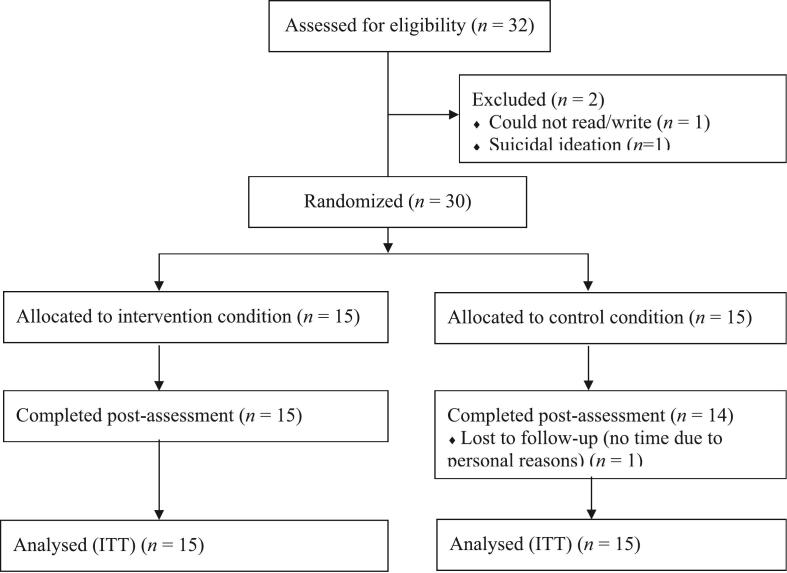
Flow diagram of participants in the study.

Fig. 1 illustrates the flow of participants throughout the trial. Demographic and loss characteristics (Table 1, Table 2) and baseline outcome measures (Table 4) did not differ significantly between the groups except for WHO-5, where the intervention group had a higher baseline score.

**Table 1:** Demographic information*.*

Demographics		Intervention group (*n* = 15)	Control group (*n* = 15)	Comparison
Age, *M* (*SD*)		42.40 (15.12)	41.73 (10.92)	*t*(28) = 0.14, *p* = 0.89
Age range		22.00–73.00	27.00–61.00	
Gender				*χ*^*2*^(1) = 0.54, *p* = 0.46
	Female	7 (46.7)	9 (60.0)	
	Male	8 (53.3)	6 (40.0)	
Marital status				*χ*^*2*^(4) = 4.56, *p* = 0.34
	Single/Never married	2 (13.3)	4 (26.7)	
	Married	11 (73.3)	7 (46.7)	
	Separated	1 (6.7)	0 (0)	
	Divorced	0 (0)	1 (6.7)	
	Widowed	1 (6.7)	3 (20.0)	
Highest education				*χ*^*2*^(4) = 3.60, *p* = 0.46
	Obligatory school	7 (46.7)	3 (20.0)	
	Apprenticeship	1 (6.7)	1 (6.7)	
	High school	1 (6.7)	4 (26.7)	
	University	2 (13.3)	3 (20.0)	
	Other	4 (26.7)	4 (26.7)	
Current Professional Situation				*χ*^*2*^(3) = 3.97, *p* = 0.26
	Employed	5 (33.3)	3 (20.0)	
	Unemployed	8 (53.3)	12 (80.0)	
	Retired	2 (13.3)	0 (0)	
Years in Switzerland, *M* (*SD*)		5.07 (3.32)	4.95 (3.01)	*t*(28) = 0.10, *p* = 0.92
Years in Switzerland, range		0.50–10.00	0.75–9.00	
Migration status (residence permit)				*χ*^*2*^(2) = 1.30, *p* = 0.52
	B	8 (53.3)	6 (40.0)	
	F	4 (26.7)	7 (46.7)	
	N	3 (20.0)	2 (13.3)	

Note: B = Permit B (recognized refugees); F = Permit F (provisionally admitted refugees), N = Permit N (permit for asylum-seekers).

**Table 2:** Loss-related variables.

Loss-related variables		Intervention group (*n* = 15)	Control group (*n* = 15)	Comparison
Relationship to lost loved one				*χ*^*2*^(7) = 7.48, *p* = 0.38
	Parent	3 (20.0)	3 (20.0)	
	Child	2 (13.3)	1 (6.7)	
	Sibling	4 (26.7)	3 (20.0)	
	Spouse	0 (0)	2 (13.3)	
	Cousin	2 (13.3)	2 (13.3)	
	Uncle	3 (20.0)	1 (6.7)	
	Nephew	1 (6.7)	0 (0)	
	Friend	0 (0)	3 (20.0)	
Gender of lost loved one				*χ*^*2*^(1) = 0.24, *p* = 0.62
	Female	2 (13.3)	3 (20.0)	
	Male	13 (86.7)	12 (80.0)	
Time since loss (years), *M* (*SD*)		4.91 (5.05)	6.37 (4.81)	*t*(28) = −0.81, *p* = 0.42
Time since loss (years), range		0.40–18.00	0.25–17.00	
Age of lost loved one, *M* (*SD*)		38.53 (20.12)	38.93 (22.55)	*t(*28) = −0.05, *p* = 0.96
Age of lost loved one, range		1.00–80.00	0.01–72.00	
Type of loss				*χ*^*2*^(4) = 3.62, *p* = 0.46
	War/violent	4 (26.7)	8 (53.3)	
	Illness	9 (60.0)	6 (40.0)	
	Accident/natural disaster	1 (6.7)	1 (6.7)	
	Natural death	1 (6.7)	0 (0)	
In Therapy				*χ*^*2*^(1) = 0.24, *p* = 0.62
	Yes	2 (13.3)	3 (20.0)	
	No	13 (86.7)	12 (80.0)	

### 3.2 Feasibility and acceptability

#### 3.2.1 Attrition, treatment adherence, and missing data

**Fig. 2:**
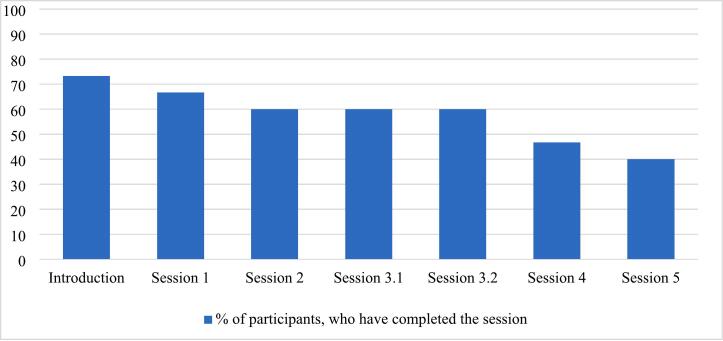
Participants completing each session (*n* = 15).

Attrition was minimal, with no participants withdrawing from the intervention group, while one participant in the control group was lost to post-assessment. Fig. 2 illustrates the number of participants who completed each session of the intervention. Participants in the treatment group completed an average of *M* = 3.74 (*SD* = 2.25) out of six sessions, with 40 % (*n* = 6) completing the final session. The introductory session (Session 0), which only outlines app functionality, was excluded from the count.

#### 3.2.2 Treatment satisfaction

The total scores on the CSQ-I ranged from 21 to 32, with an average score of *M* = 27.40 (*SD* = 3.66). Of the participants, 93.33 % (*n* = 14) agreed that the intervention was of high quality, based on the proportion selecting either “agree” (3) or “fully agree” (4). Additionally, 80 % (*n* = 12) felt the program met their needs, 80 % (*n* = 12) agreed that the app helped them deal with their problems more adequately, and 100 % (*n* = 15) expressed overall satisfaction with the intervention.

#### 3.2.3 Feasibility and acceptability – qualitative results

The following themes emerged from the qualitative interviews with the intervention group: Usability, delivery format, surface structure, content general, and exercises and interventions. The most significant findings will be presented in the following subchapters. Selected participant quotes can be found in Table 3.

**Table 3:** Selected participant quotes.

Topics	Sub-topics		Quotes
Usability	User satisfaction	Overall satisfaction	“This is a very nice app. It was a very nice experience and a new experience for me.”
		Would use again, recommend it to others	“The content of the app that I liked, I apply in my everyday life. Always, not just now because of the grieving situation. Because I have benefited so much. And I always do it, and I also recommend it to others and talk about it.”
	User-friendliness	Technical problems, audio	“Technical problems. Sometimes you can't listen to the audios until the end, I couldn't listen to them all the way through, or with videos, they suddenly freeze, or when I'm watching something, I can't continue. That's what I meant, not the content. Just technical problems that still need improvement.”
	User experience	Stories	“That was also good because when you read the stories of others, you can calm down a bit. That others have experienced the same or more or less the same. […] But how they deal with it, how they regain hope, that was helpful.”
			“Yes, the stories were good. Sometimes I thought […] they find joy in small things – they are content with little things. And then I thought to myself: Ah, I have that too. And then you start reflecting”
		Life goes on	“But it feels comforting that you've endured all of that. And you've picked yourself up and carried on.”
		Sadness	“No, these feelings of ‘sadness’ belong to the app and this topic.”
		More positive content	“A little more positivity. There's a section, for example, about grief and depression and how the protagonists are sad and so on, but a bit more about happiness is needed. There should always be something about happiness, always a point about happiness.”
Delivery format		Early stages of grief	“For someone who has a new injury or has had one for a year or six months, I think it's very appropriate.”
Surface structure	Vignettes	Representative	“The stories were good and fitting because we have all experienced something similar, more or less.”
		Understanding own feelings	“When I read these stories, they were able to express what they feel. That helped me a lot to express or recognize my own feelings.”
		Not intense enough	“It might be better and more helpful if there were a more difficult story, if there were some more challenging stories than this.”
	Structuration	Tempo, taking breaks	“But because they said in advance, just according to my tempo. If it's no longer good for me, or too hard – then I should take a break. That felt good.”
		Availability of chapters	“When I finished reading the first chapter, I was so motivated; I wanted to read everything. Then I had to wait – a week – until the second chapter was opened, and that was […] I was no longer motivated because I had to wait so long. […] In my opinion, it would be better if all the chapters were opened from the beginning.”
Content general		Relevance	“It is very important. Especially for Syrian refugees, because most or almost all of them have already experienced something, and that is actually important, yes.”
			“It was very helpful because these stories and the content of the app, in my opinion, are not only good for loss due to death but also for other things. Because Syrian refugees suffer from many difficulties, such as fleeing or people who still have no place, no housing, or no identification, etc.”
		Acceptability	“And above all, it addresses many aspects. There were also many parts that simply referenced things from the Quran. I thought that was great. Because last time we mentioned it, and now that it has been included, I find it very… Many are also Muslims. And that is actually very acceptable.”
		Helpful	“You understand exactly what/why you are suffering. These are not only physical but also psychological. […] When you engage with it, you understand the negative influences and the positive ones and then you look for a solution.”
		Acceptance	“It was a positive feeling that a person can continue to live after grief. Life must go on; when someone dies, life doesn't stop. Just because someone has died, you don't have to die yourself, for example, by not working or doing nothing; that makes no sense. Life must go on, and you have to accept the pain; you have to accept death.”
Interventions and exercises		Helpful	“The advantages are the exercises. If you really implement or practice these exercises. […] Especially […] the physical exercises. In my opinion, after a month of practicing them regularly, you will forget your grief or significantly reduce it.”
		Social activities	“There was an exercise or activity that helps you integrate more with the family. […] before, I was the kind of person who: Yes, I took good care of the children, school, making sure they didn't need anything. But still, I was somehow not present when we were together. And now, after this exercise, I realize it has gotten better. It's not just about what they need, but also what they need psychologically. Playing with them, talking, doing something together.”
		Self-compassion	“I now know that my self also has a right! I don't always have to give, give, give. Sometimes I have to/(look after myself). Yes, exactly. I used to think that it was selfish of me, but no.”
			“There was an exercise that said: ‘If you want to comfort a friend. Your friend is in your situation, and you would comfort them or talk to them. What would you do?’ […] That was so good. That was very helpful.”
		Tree of legacy and breathing exercise	“The one exercise, the memory tree exercise, was very good for the mind and makes you feel good. And the breathing exercise was very good for the body.”
		Too many exercises	“Actually, all the exercises are good. It doesn't depend on the exercises themselves. There are just so many, and that's why I said that people can't implement all of them. Maybe they can use about half of the exercises. So, if you could reduce it to about half the number of exercises.”

##### 3.2.3.1 Usability

The theme usability refers to user satisfaction, user-friendliness and user experience. Regarding user satisfaction, all participants expressed overall satisfaction with the IBI (15; 100 %). Most stated they would use the app again (13; 87 %) and had recommended or would recommend it to others (13; 87 %). However, one participant said he would not recommend the app (1; 7 %), and two raised concerns: one found there were too many exercises (1; 7 %), another suggested financial support should be provided and preferred a guided approach rather than dealing with emotions alone (1; 7 %). Regarding the app's user-friendliness, 11 participants (75 %) found it generally easy to use. They highlighted clear guidance and navigation through the exercises and chapters (3; 20 %), useful reminder notifications from the planner (1; 7 %), and the option to write within the app (2; 13 %). A majority reported difficulties or suggestions regarding the app's usability (9; 60 %). Six users (40 %) mentioned technical problems (e.g., being logged out of the app, not being able to play audios, etc.), while suggestions included adding the option to share one's own story with others (1; 7 %), adding more options to write (1; 7 %), adding the option to draw instead of write for certain exercises (1; 7 %), and providing the text as audio for people who cannot read (1; 7 %). The theme user experience captured different thoughts and feelings that resulted from the use of the app. Thoughts included that reading or hearing about similar stories of others through the app gave hope and perspective and was useful in dealing with one's own grief (4; 27 %), that death must be accepted and life goes on (2; 13 %), and that taking the time to reflect one's own story and experience is important to understand and come to term with one's grief (1; 7 %). Participants' feelings included sadness and grief (7; 47 %), feeling more relaxed (5; 33 %), compassion from and for others (3; 20 %) and hope for the future (2; 13 %). Several participants (6; 40 %) mentioned mixed feelings of intense sadness and positive feelings, with this being part of and important for the grieving process. Eight participants (53 %) suggested modifications to the content including adding more positive content (3; 20 %) and adding additional information/support through institutions, church or community (4; 27 %).

##### 3.2.3.2 Delivery format

Several advantages specifically related to the delivery via app were mentioned including the flexibility of use (2; 13 %) and the use of notifications (1; 7 %). A majority reported barriers to use for them or others (8; 53 %) including issues of accessibility for people with low education or literacy levels (2; 13 %), lack of time (3; 20 %), technical issues or low technical literacy (3; 20 %). Furthermore, several responses related to differences in usefulness/ease of use for different groups. Two participants (13 %) mentioned that the app could be especially useful in the early stages of grief, while others (4; 27 %) thought the app may be less suitable for individuals experiencing more severe grief (e.g., child-loss, traumatic losses).

##### 3.2.3.3 Surface structure

The theme surface structure refers to feedback regarding the IBI's audiovisual aspects as well as the language and structuration of content. The language received only positive feedback with participants praising its clarity and comprehensibility (14; 93 %). Videos were appreciated by users as being helpful (4; 27 %), more so than audios due to their conciseness (2; 13 %). One person (7 %) suggested that a positive outlook should be added to the videos (e.g., what helped them feel better). All participants provided positive feedback regarding the illustrations. They were found particularly helpful in clarifying the text (2; 13 %), to underline the message and feelings conveyed (4; 27 %) and representative (1; 7 %). One person (7 %) recommended showing both women with and without headscarves for diversity.

The audio-format was positively received by several participants (5; 33 %). Three participants (20 %) found the audios too long and suggested shortening them. The vignette stories were positively received by most participants (14; 93 %). They were seen as generally positive and representative (8; 53 %), found to be calming to read and helpful to recognize and understand one's own feelings (7; 47 %), and were seen as inspirational and motivating (4; 27 %). Six participants (40 %) pointed out difficulties or proposed modifications to the vignettes. For instance, two people (13 %) thought the vignettes induced too much sadness and one thought more positivity should be added (7 %), while two others (13 %) thought the grief story was not “intense” enough and should include more traumatic losses to be more representative.

Positive feedback on the structuration of the app was given by seven participants (47 %). It was thought to be well-structured (2; 13 %), the length of the content appropriate (5; 33 %), the presentation clear (1; 7 %) and the reminders that participants could take a break whenever needed were seen as helpful (1; 7 %). Four participants (27 %) mentioned individual points regarding difficulties or modification of the structuration including finding the chapters too long (1; 7 %), suggesting that the subsequent chapter be made available immediately after finishing the previous one (1; 7 %), and placing the more difficult/sad chapters at the beginning of the app (1; 7 %).

##### 3.2.3.4 Content general

All participants provided positive feedback regarding the content of the IBI. Users reported general satisfaction with the topics (e.g., social relationships, plans for the future) and content (11; 73 %), felt the content was culturally acceptable (14; 93 %) and relevant (10; 67 %), and found it useful (11; 73 %). Participants outlined that the inclusion of religion helped make the content more acceptable (3; 20 %).

Furthermore, it was reported that the content helped to understand and deal with one's own feelings better and to understand the connection between physical and psychological complaints (3; 20 %). Others stated that it helped them accept the loss and look forward (3; 20 %).

##### 3.2.3.5 Interventions and exercises

Most participants (13; 87 %) reported positive feedback relating to the interventions and exercises. The exercises were mentioned as a main advantage of the IBI and being experienced as helpful for instance in reducing grief symptoms and feeling more relaxed (12; 80 %). Several participants highlighted exercises regarding social activities as particularly helpful (3; 20 %), others particularly found the mindfulness and breathing exercises to be helpful when feeling sad or distressed and reported using them often (7; 47 %). Three participants (20 %) appreciated that writing was included in several exercises, and two (13 %) reported that exercises for self-compassion helped them especially. Several participants mentioned that they appreciated the tree of legacy exercise where they reflected about their loved one and their relationship (3; 20 %). Several suggestions for modifications to the content of the exercises were made including adding more exercises in general (2; 13 %), and specifically to improve self-worth or to share one's own grief story with other users. Another user (7 %) wished for a reduction in the number of exercises.

### 3.3 Preliminary efficacy

Table 4 presents the means, standard deviations, and effect sizes for the baseline and post-assessment outcome measures. Table 5 outlines the fixed effects results from the primary models (mixed linear models).

**Table 4:** Means, SDs, effect sizes (Cohen's d), baseline comparison for outcomes.

		Baseline			Post-assessment			Effect size (Within, baseline-post)	Effect size (Between, post)	Baseline comparison
		*M*	*SD*	*n*	*M*	*SD*	*n*	*d* [95 % CI]	*d* [95 % CI]	
IPGDS	Intervention	100.13	27.77	15	87.27	27.05	15	−0.44 [−0.72, −0.16]	−0.51 [−1.27, 0.24]	*t*(28) = −0.68, *p* = 0.50
	Control	108.19	36.81	15	103.86	35.45	14	−0.18 [−0.43, 0.06]		
PHQ-9	Intervention	12.13	8.48	15	10.80	7.05	15	−0.16 [−0.43, 0.11]	−0.22 [−0.99, 0.54]	*t*(28) = −0.69, *p* = 0.50
	Control	14.23	8.17	15	12.50	8.06	14	−0.31 [−0.67, 0.06]		
PCL-5	Intervention	34.67	16.43	15	27.00	18.35	15	−0.43 [−0.79, −0.08]	−0.53 [−1.3, 0.25]	*t*(28) = −0.80, *p* = 0.43
	Control	39.81	18.75	15	36.71	18.62	14	−0.22 [−0.59, 0.14]		
GAD-7	Intervention	11.47	6.81	15	9.27	6.18	15	−0.34 [−0.71, 0.04]	−0.54 [−1.32, 0.24]	*t*(28) = −0.77, *p* = 0.45
	Control	13.22	5.58	15	12.57	6.07	14	−0.19 [−0.55, 0.18]		
WHODAS 2.0	Intervention	19.44	13.18	15	16.60	11.82	15	−0.22 [−0.47, 0.02]	0.08 [−0.68, 0.84]	*t*(28) = 0.77, *p* = 0.45
	Control	15.91	12.07	15	15.57	14.28	14	−0.05 [−0.29, 0.19]		
WHO-5	Intervention	12.40	5.59	15	14.73	5.97	15	0.40 [−0.15, 0.96]	1.02 [0.21, 1.83]	*t*(28) = 2.56, *p* = 0.02
	Control	7.80	4.13	15	9.21	4.79	14	0.33 [−0.18, 0.84]		
PMLD	Intervention	32.53	10.12	15	29.47	13.03	15	−0.26 [−0.75, 0.23]	−0.3 [−1.06, 0.47]	*t*(28) = 0.10, *p* = 0.92
	Control	32.14	12.18	15	33.50	14.32	14	0.12 [−0.42, 0.65]		
MSPSS	Intervention	58.27	17.45	15	62.93	14.25	15	0.28 [−0.02, 0.57]	0.32 [−0.45, 1.09]	*t*(28) = 0.38, *p* = 0.71
	Control	55.64	20.32	15	57.71	18.41	14	0.10 [−0.17, 0.37]

**Table 5:** Results of linear mixed model analyzes.

Outcome	Time				Group x Time Interaction			
	*B*	*SE*	95 % CI	*p*	*B*	*SE*	95 % CI	*p*
IPGDS	−4.31	4.06	−12.63, 4.01	0.30	−8.56	5.64	−20.14, 3.02	0.14
PHQ-9	−1.72	1.31	−4.40, 0.96	0.20	0.39	1.82	−3.35, 4.12	0.83
PCL-5	−3.57	3.09	−9.92, 2.78	0.26	−4.10	4.31	−12.94, 4.75	0.35
GAD-7	−0.82	1.16	−3.20, 1.56	0.49	−1.38	1.61	−4.69, 1.93	0.40
WHODAS 2.0	−0.39	1.54	−3.54, 2.77	0.80	−2.01	2.14	−6.40, 2.38	0.36
WHO-5	1.46	1.34	−1.29, 4.21	0.29	0.87	1.87	−2.97, 4.72	0.64
PMLD	2.10	3.20	−4.46, 8.66	0.52	−5.17	4.46	14.32, 3.98	0.26
MSPSS	2.21	2.64	−3.21, 7.63	0.41	2.66	3.68	−4.89, 10.21	0.48

#### 3.3.1 Primary outcome

A medium between-group effect was observed on the IPGDS, favoring the treatment group over the control (Table 4). However, the main model showed no significant group-by-time interaction (Table 5). Exploratory analyses included time, group, and sex, along with their interactions, in the model and revealed significant interaction effects, including a three-way interaction (time × group × sex; *B* = 35.44, *SE* = 9.73, *p* = 0.001) and two-way interactions (time × group: *B* = −28.09, *SE* = 7.28, *p* = 0.007; time × sex: *B* = −17.75, *SE* = 7.12, *p* = 0.02). A simple slope analysis was conducted to further probe the three-way interaction (time × group × sex). Among males, the effect of group on time was significant (*B* = 21.12, *SE* = 4.53, *p* = 0.0001), indicating that males in the intervention group experienced significantly less grief at the second time point compared to the first. Among females, the effect of group on time was not significant (*B* = 3.43, *SE* = 4.84, *p* = 0.49), suggesting no meaningful change in grief over time for females in the intervention group.

#### 3.3.2 Secondary outcomes

Across secondary outcomes, no significant group-by-time interactions were observed. Small post-intervention between-group effect sizes favoring the treatment group were found for the PHQ-9, PMLD, and MSPSS; medium effects for the PCL-5 and GAD-7; and a large effect for the WHO-5. Negligible effects were observed for the WHODAS 2.0. Complete details of the effect sizes and statistical analyses are provided in Table 3, Table 4.

## 4 Discussion

The aim of this mixed-methods pilot RCT was to assess the acceptability, feasibility, and preliminary efficacy of a culturally adapted IBI targeting bereaved Syrian refugees in Switzerland. To the best of our knowledge, this is the first study to evaluate a culturally adapted IBI for bereaved individuals in general, and for Syrian refugees specifically. This study found that the culturally adapted IBI was feasible and acceptable, as evidenced by low dropout rates (3.3 %), an adherence rate of 40 %, and high participant satisfaction (mean CSQ-I score: 27.4/32). Qualitative feedback highlighted the intervention's usability and cultural relevance. Exploratory analyses suggested moderate reductions in grief severity and other outcomes, although no significant effects were observed.

### 4.1 Summary of findings

Findings revealed that the tested IBI was found to be acceptable and feasible within the target group. One notable indicator of feasibility was the exceptionally low dropout rate, with only one participant (3.3 %) from the control group withdrawing. Dropout rates in other culturally adapted IBIs are typically higher. For example, Lindegaard et al. (2021) reported a 39 % dropout rate in an IBI for Arabic speakers in Sweden. A recent review of IBIs for refugees found dropout rates ranging from 2.9 % to 80 %, with higher rates being common (El-Haj-Mohamad et al., 2023).

In terms of adherence, 40 % of participants in the intervention group completed the full IBI. This rate aligns with typical adherence levels in other IBIs, which range from 32 % to 46 % (Berger et al., 2017; Cuijpers et al., 2022; Kazlauskas et al., 2020). Qualitative findings highlighted potential barriers to app use, including accessibility challenges for individuals with low literacy or education levels, time constraints, and technical issues. Additionally, some participants suggested the app might be more suitable for early stages of grief or less severe grief. The app's flexibility in allowing selective engagement with content may also have contributed to non-completion. Future research, such as dismantling studies, could help identify which components of the app are most effective for different subgroups. El-Haj-Mohamad et al. (2023) reported a mean adherence rate of 37.51 % in IBIs for refugees, indicating that this IBI performed comparably well in terms of adherence.

A key challenge in culturally adapted IBIs is maintaining participant engagement, with more research needed to address this in IBIs (El-Haj-Mohamad et al., 2023). Many studies lack qualitative evaluations (e.g., Lindegaard et al., 2021), limiting understanding of disengagement. This study, however, used a mixed-methods approach, providing deeper insights into the low dropout rate and 40 % adherence. The success was largely attributed to thorough cultural adaptation and sensitive execution, which, as shown by Naeem et al. (2019), can enhance adherence by aligning interventions with participants' values and needs.

A key part of the adaptation was making the treatment goals culturally relevant, likely raising participants' expectations of success, which has been linked to better adherence in IBIs (Kazlauskas et al., 2020) and lower dropout rates (Vöhringer et al., 2020). Introducing these adapted goals early in the first session likely increased the intervention's credibility, improving adherence. While this assumption is supported by prior research, we do not have direct confirmation of this in our results. Additionally, addressing the diversity within the target population was crucial, as neglecting differences can harm adherence (El-Haj-Mohamad et al., 2023; Heim et al., 2024). This careful adaptation likely reduced dropout and enhanced engagement.

Qualitative findings emphasized the role of interactive elements, such as video testimonials and vignette stories, in maintaining participant interest. These features were praised for their motivational impact, consistent with research showing that interactive components boost engagement in IBIs (Harper Shehadeh et al., 2020). Participants also appreciated the clear, linear structure, which enhanced usability. In contrast, Röhr et al. (2021) suggested that unclear guidance in their app led to lower engagement. The structured design of this IBI likely improved adherence.

While this IBI met many participant needs, further customization may be required for subgroups with varying educational or literacy levels, such as adding a text as audio option or the option of drawing instead of writing, with the former planned for the final app version. Usability testing, including “think aloud” sessions, resolved many technical issues from an earlier phase, likely improving navigation (Aeschlimann et al., 2024a). However, some technical challenges persisted, creating barriers, similar to findings in other studies where technical problems led to frustration and dropout (Heim et al., 2021b). Resolving the remaining issues is critical prior to final implementation. Despite these issues, participants generally found the app user-friendly with clear language.

The IBI demonstrated high acceptability, with a mean CSQ-I score of 27.4 out of 32, slightly higher than other culturally adapted IBIs. For instance, an online mindfulness intervention for international students scored 25.4 (Balci et al., 2023), and an internet intervention for Indonesian students scored 25.8 (Rahmadiana et al., 2021). An IBI targeting sleep in refugees scored 24.0 (Spanhel et al., 2022). Although minor, these differences suggest the cultural adaptation of this IBI may have enhanced participant satisfaction compared to other IBIs, as evidenced by the higher CSQ-I scores reported in this study. In contrast, Efinger et al. (2022) found 30 % dissatisfaction due to complex content and technical issues. Here, participants appreciated the clarity and cultural relevance, likely boosting satisfaction.

The high acceptability and satisfaction levels likely stemmed from the extensive cultural adaptation, as previous research shows cultural adaptation improves acceptability (Arundell et al., 2021). In qualitative interviews, all participants reported overall satisfaction, 93 % felt it was well culturally adapted, and most would use or recommend the IBI. Participants particularly appreciated topics like social support and future orientation, with the inclusion of religious content enhancing cultural acceptability. While the inclusion of religious content was intended to enhance cultural acceptability, we acknowledge the diversity within Syrian communities regarding religiosity. The intervention was designed to be inclusive of various religious perspectives (e.g., Muslim, Christian, Druze) based on input from participants during the development phase. However, less religious individuals might resonate differently with these elements. Nonetheless, religiosity has been identified as a culturally significant factor in bereaved Arabic-speaking populations, underscoring the importance of offering flexible, customizable interventions to accommodate varying beliefs and preferences (Lechner-Meichsner and Comtesse, 2022).

Qualitative findings indicated participants found the IBI helpful in reducing grief, supported by moderate, though not statistically significant, reductions in grief severity. While this pilot RCT was not powered to detect significant effects, these trends align with prior RCTs of IBIs targeting grief, which report moderate effects (Hedge's *g* = 0.54; Wagner et al., 2020; Zuelke et al., 2021). Similarly, moderate effect sizes were reported in a pilot RCT conducted with Syrian refugees in Switzerland using PM+, a face-to-face intervention designed to reduce mental distress in conflict-affected populations (Spaaij et al., 2022). These consistent findings highlight the value of a fully powered RCT to assess the IBI's efficacy. Although exploratory, the results of the simple slope analysis suggest that the intervention may have been more beneficial for men. This difference could possibly be due to societal expectations in Syrian culture regarding emotional expression. As noted by Lechner-Meichsner and Comtesse (2022), these expectations often dictate that women are encouraged to show their emotions, while men are discouraged, which can hinder their ability to cope with loss. The low-threshold nature of the intervention likely mitigated this barrier, providing men with a rare opportunity to engage with coping strategies in a culturally acceptable way. In contrast, women may have relied on other emotional or interpersonal coping mechanisms outside of the intervention. These findings also suggest potential implications for future adaptations of the intervention to better address gender-specific needs. However, these results should be interpreted cautiously given the small sample size and would need to be examined in a fully powered RCT.

### 4.2 Limitations

This study has several limitations that should be acknowledged. The small sample size limits statistical power, which is typical for pilot RCTs focused on assessing feasibility and acceptability rather than generalizing results. While preliminary evidence was collected, subgroup analysis—such as the intervention's suitability for those with traumatic losses, or longer/shorter time since loss—was not possible. These factors, noted in the qualitative feedback, are known to impact grief severity in the literature (Djelantik et al., 2020b). Baseline differences, such as in WHO-5 well-being, were accounted for within the linear mixed-effects model, minimizing potential bias.

The study's strong adherence rates may be partly due to in-person evaluations and compensation, potentially limiting the generalizability of adherence in fully remote settings. In-person assessments could also have inflated treatment satisfaction due to social desirability bias, as participants may have felt pressure to report more positive experiences.

Another potential source of bias involves the interpreters, some of whom had prior professional relationships with participants through their work as translators. While interpreters were instructed to maintain neutrality, these prior interactions may have influenced participant responses. Future studies should aim to ensure interpreters are unacquainted with participants to minimize this risk.

Additionally, the 5-week intervention duration was relatively short, with some participants noting in qualitative feedback that a longer period would have been more beneficial; future studies may consider extending the intervention.

Interviews relied on translation, potentially affecting the depth and accuracy of feedback. Not all participants completed every chapter, limiting comprehensive feedback on the full intervention.

Finally, Ramadan coincided with data collection, posing challenges for recruitment and result interpretation. Fasting-related fatigue may have influenced assessment quality, and the heightened emotional intensity of the holiday may have impacted grief experiences, as noted in the literature (Carr et al., 2014; Wilson et al., 2021). Thus, the timing of data collection may have affected participant responses and the perceived efficacy of the intervention.

## 5 Conclusion

This study aimed to evaluate the feasibility, acceptability, and preliminary efficacy of a newly developed IBI for bereaved adult Syrian refugees. The findings suggest that the intervention is both feasible and acceptable. While the preliminary results show promising effects on primary and secondary outcomes, they were not statistically significant, and further evaluation in a fully powered RCT is needed. Nonetheless, this culturally adapted IBI shows potential to provide valuable support to bereaved Syrians, offering a possible solution to help address the treatment gap.

## CRediT authorship contribution statement

AA, EH, MT, RS, MA, AM and CK conceived the study. AA designed the work. AA, NM, and FH acquired the data, with analysis and data interpretation by AA. AA drafted the manuscript, which was substantively revised by AA, EH, CK, MT, NM, FH, RS, MA, and AM. All authors read and approved the final manuscript.

## Declaration of Generative AI and AI-assisted technologies in the writing process

During the preparation of this work the author(s) used ChatGPT in order to improve the grammar and avoid spelling mistakes. After using this tool/service, the author(s) reviewed and edited the content as needed and take(s) full responsibility for the content of the publication.

## Funding

The first author is funded by a doctoral scholarship from the Digital Society Initiative (DSI) of the University of Zurich. The overall project was funded by the Humanitarian Foundation of the SRC.

## Declaration of competing interest

The authors declare the following financial interests/personal relationships which may be considered as potential competing interests: naïs Aeschlimann reports financial support was provided by Swiss Red Cross. Anaïs Aeschlimann reports financial support was provided by Digital Society Initiative University of Zurich. If there are other authors, they declare that they have no known competing financial interests or personal relationships that could have appeared to influence the work reported in this paper.

## Appendix A Supplementary data

Appendix A Image 1

Appendix B Full description of measures Image 2

Appendix C Interview guide (only for T1 in the intervention group) Image 3
