# Supplementary material for: Cultural adaptation of a self-help app for grieving Syrian refugees in Switzerland. A feasibility and acceptability pilot-RCT
Source: Internet Interv. 2025 Jan 18;39:100800. doi: 10.1016/j.invent.2025.100800 (PMC11788602; doi:10.1016/j.invent.2025.100800)
Supplement: Supplementary file 2 — Appendix B Full description of measures [file mmc2.pdf]

Appendix B: Full description of measures

Feasibility and acceptability

Treatment satisfaction was assessed using the Client Satisfaction Questionnaire for Internet Interventions (CSQ-I; Boß et al., 2016). This questionnaire comprises eight items (α = 0.83), each rated on a scale from 1 (disagree) to 4 (fully agree), yielding sum scores between 8 and 32, with higher scores indicating greater satisfaction. Key areas of interest included ratings on the perceived quality, relevance and usefulness of the intervention, as well as overall satisfaction with the treatment, specifically in questions one, three, six, and seven.

Feasibility and acceptability – qualitative evaluation. A brief semi-structured interview was developed based on Kallio et al. (2016), background literature, the RECAPT criteria, and results from previous project phases (Aeschlimann et al., 2024). Feedback focused on the feasibility, acceptability, and usefulness of the IBI, including design, content, and interactive elements, with suggestions for refining unclear or irrelevant aspects (see Appendix B). Two pilot interviews were conducted to refine the guide.

Adherence was measured through the percentage of the app that was completed. Participants were asked at T1 to show the interviewer the progress in the app to determine how many sessions and subchapter they had completed.

Preliminary efficacy

Grief severity. The International ICD-11 Prolonged Grief Disorder Scale (IPGDS; Killikelly & Maercker, 2017) was used, featuring 33 items (α = 0.96) rated on a 5-point scale from 1 (not at all) to 5 (always). Higher scores indicated greater grief severity (total possible score = 32-160).

Secondary outcomes

Depression symptoms. The Patient Health Questionnaire-9 Item (PHQ-9; Kroenke & Spitzer, 2002) was used, with 9 items (α = 0.93) scored from 0 (never) to 3 (practically every day). Higher scores indicate greater depressive severity (total possible score = 0-27).

PTSD symptoms. The PTSD Checklist 5 (PCL-5; Price et al., 2016) with 20 items (α = 0.94) was employed, with responses scored from 0 (not at all) to 4 (extremely). Higher scores reflect more severe PTSD symptoms (total possible score = 0-80).

Anxiety symptoms. The Generalized Anxiety Disorder Screener (GAD-7; Spitzer et al., 2006) was used, with 7 items (α = 0.91) scored from 0 (not at all) to 3 (nearly every day). Higher scores indicate greater anxiety severity (total possible score = 0-21).

Health and disability levels. The 12-item (α = 0.94) version of the WHO Disability Assessment Scale 2.0 (WHODAS 2.0; Üstün et al., 2010) was employed, with items rated on a scale from 0 (none) to 4 (extreme or cannot do). Higher scores indicate higher disability levels (total possible score = 0-48).

Psychological well-being. The WHO-5 Wellbeing Index (Bech et al., 2003) was used, with 5 items (α = 0.89) rated from 0 (at no time) to 5 (all the time). Higher scores indicate better well-being (total possible score = 0-25).

Further outcomes

PMLD. The Post Migration Living Difficulties Scale (PMLD; Schick et al., 2016) was used, with 17 items (α = 0.82) rated from 0 (not a problem) to 4 (very serious problem). Higher scores indicate more difficulties (total possible score = 0-68).

Perceived Social Support. The Multidimensional Scale of Perceived Social Support (MSPSS; Zimet et al., 1988) was employed, with 12 items (α = 0.91) rated from 1 (very strongly disagree) to 7 (very strongly agree). Higher scores indicate greater perceived social support (total possible score = 12-84).

Sociodemographic Information. Collected data included sex, age, ethnicity, marital status, education, work situation, immigration status, time in Switzerland, and loss-related characteristics such as the relationship to the deceased, cause of death, and current psychotherapeutic treatment status.
