# Supplementary material for: Cultural adaptation of a self-help app for grieving Syrian refugees in Switzerland. A feasibility and acceptability pilot-RCT
Source: Internet Interv. 2025 Jan 18;39:100800. doi: 10.1016/j.invent.2025.100800 (PMC11788602; doi:10.1016/j.invent.2025.100800)
Supplement: Supplementary file 3 — Appendix C Interview guide (only for T1 in the intervention group) [file mmc3.pdf]

Interview guide (only for T1 in the intervention group)

(English translation)

Introduction:

Thank you very much for making yourself available for this interview. You have been using the Sui app on grief to support grief for Syrian refugees in Switzerland for the past 5 weeks. Now we would like to ask for your feedback to see if using the app was acceptable, feasible and useful for you. We would like your help to know how we can adapt the app even more to the needs of Syrian refugees. We would like to know what you liked and disliked about the app, what problems you may have encountered and what suggestions you have for improving the app. Your valuable feedback and that of other testers will be incorporated into the app in the next step. If you do not want to answer a question, that is of course fine and we will skip that question. If you need a break, you can let us know at any time. The interview today will take a maximum of 1 hour.
Now let's start the interview.

Interview

- Now let's start the interview.
- I would like to ask you a few questions about your opinion of the app.

Topic	Formulation	Notes
**1. General impression**	*What is your first or general impression of the app? How do you think other Syrian refugees would like the app?*	
	In your opinion, what are the advantages and disadvantages of the app?<br>*How could we deal with the disadvantages?*	
	What was the biggest problem or difficulty you had with the app?<br>*(What do you see as the biggest problem or difficulty regarding the use of the app for other Syrian refugees?)*<br>*How could we deal with this?*	
	Did you find the app user-friendly?<br>*If not, what bothered you the most? And how could we improve it?*	
	How did you feel when using the app?	

**2. Design, illustrations, vignettes and (audio-) exercises**	*What do you like about the design and illustrations and what don't you like so much? How could this be improved?*	
	Were the vignettes helpful when working through the chapters? What was helpful/not helpful?<br>*If not, what can we do to make this better?*	
	How do you like the exercises and audio exercises in the app?	
	Which exercises and audio exercises were particularly helpful and which were not?<br>*What can we do to make this better?*	
	Do you think that something important is still missing with regard to the design, the illustrations and the (audio) exercises?<br>*If so, what?*	
**3. Content, topics and usefulness**	*How do you like the content and topics of the app?*<br>*What do you particularly like and what don't you like so much?*<br>*If you don't like something, what can we do to make it better?*	
	Is everything clear and understandable?<br>*Were there any linguistic or comprehension obstacles that made it difficult to work through the chapter(s)?*	
	Are the contents and topics acceptable for Syrian refugees?	

	Or can Syrian refugees have a negative reaction to this content, such as feeling stressed, annoyed or offended?	
	Are the content and topics relevant for Syrian refugees?	
	How can we change the content and themes of the app to make it more relevant and acceptable to Syrian refugees?	
	How useful did you find the app's content and topics as support for your grief?	
	What content and topics did you find particularly useful in supporting your grief?<br>*Which not?*	
	What could be improved to make the content even more useful for Syrian refugees?	
	Do you have the feeling that one or more important topics are not being addressed? If so, which ones?	
**4. Adherence**	*Have you worked through all the chapters in the app?*	
	If so, how easy or difficult did you find it to complete the chapters?<br>*If not, which ones have you not worked through?*	

	Can you think of any reasons why you did not work on chapter(s) XX?	
	What do you think could be done differently in the chapter(s)?	
	What could be done to encourage Syrian refugees to use the app for longer?	
	Would you use the app again if you needed help?	
	Would you recommend this app to a friend or family member if they needed similar help?	
**5. Additions**	*Is there anything else you would like to tell us about the app that we haven't discussed yet?*	
	Have you noticed anything else?	
	Is there anything else you would like to tell us about the app that we haven't discussed yet?	
	Thank you very much for your time and your answers, you have helped us a lot! If you have any further questions or concerns in the future, please do not hesitate to to contact us at any time.	
